# 4094 Structural Determinants of Immunogenicity for Peptide-Based Immunotherapy

**DOI:** 10.1017/cts.2020.92

**Published:** 2020-07-29

**Authors:** Jason Devlin, Jesus Alonso, Grant Keller, Sara Bobisse, Alexandre Harari, Brian Baker

**Affiliations:** 1 Indiana University School of Medicine; 2 University of Notre Dame; 3 UNIL CHUV, Ludwig Institute for Cancer Research, Switzerland

## Abstract

OBJECTIVES/GOALS: Neoantigen vaccine immunotherapies have shown promise in clinical trials, but identifying which peptides to include in a vaccine remains a challenge. We aim to establish that molecular structural features can help predict which neoantigens to target to achieve tumor regression. METHODS/STUDY POPULATION: Proteins were prepared by recombinant expression in *E. coli* followed by *in vitro* refolding. Correctly folded proteins were purified by chromatography. Affinities of protein-protein interactions were measured by surface plasmon resonance (SPR) and thermal stabilities of proteins were determined by differential scanning fluorimetry. All experiments were performed at least in triplicate. Protein crystals were obtained by hanging drop vapor diffusion. The protein crystal structures were solved by molecular replacement and underwent several rounds of automated refinement. Molecular dynamics simulations were performed using the AMBER molecular dynamics package. RESULTS/ANTICIPATED RESULTS: A T cell receptor (TCR) expressed by tumor-infiltrating T cells exhibited a 20-fold stronger binding affinity to the neoantigen peptide compared to the self-peptide. X-ray crystal structures of the peptides with the major histocompatibility complex (MHC) protein demonstrated that a non-mutated residue in the peptide samples different positions with the mutation. The difference in conformations of the non-mutated residue was supported by molecular dynamics simulations. Crystal structures of the TCR engaging both peptide/MHCs suggested that the conformation favored by the mutant peptide was crucial for TCR binding. The TCR bound the neoantigen/MHC with faster binding kinetics. DISCUSSION/SIGNIFICANCE OF IMPACT: Our results suggest that the mutation impacts the conformation of another residue in the peptide, and this alteration allows for more favorable T cell receptor binding to the neoantigen. This highlights the potential of non-mutated residues in contributing to neoantigen recognition.

